# Transcriptomic Analysis of Genes Associated with Stinger Development at Different Life Stages of *Apis mellifera*

**DOI:** 10.3390/ijms251910746

**Published:** 2024-10-06

**Authors:** Shiwen Zhou, Juan Zhang, Zhenhui Yang, Yunxi Fu, Yu Lai, Xueling Xu, Ruixin Xu, Yang Lü, Zhiguo Li, Ping Zhao, Songkun Su, Hongyi Nie

**Affiliations:** 1 College of Bee Science and Biomedicine, Fujian Agriculture and Forestry University, Fuzhou 350002, China; zsw19980505@163.com (S.Z.); 17370823565@163.com (J.Z.); yangzhenhuikkk@163.com (Z.Y.); fuyxfafu@foxmail.com (Y.F.); yu@fafu.edu.cn (Y.L.); xv_ruixin@163.com (R.X.); 6220623004@fafu.edu.cn (Y.L.); zhiguo.li@fafu.edu.cn (Z.L.); 2 State Key Laboratory of Resource Insects, Biological Science Research Center, Southwest University, Chongqing 400715, China; 3 Mudanjiang Branch of Heilongjiang Academy of Agricultural Sciences, Mudanjiang 157000, China

**Keywords:** *Apis mellifera*, stinger, morphological observation, transcriptome, highly expressed genes, *Dll*, RNAi, tip bending

## Abstract

Stingers, evolved from ovipositors, are an important defense organ for the *Apidae*, *Vespidae*, and *Formicidae* species. However, the molecular mechanism of stinger development remains unclear. Here, we show that the earliest time point for the appearance of stingers in *Apis mellifera* is at the 1-day-old worker pupal stage based on morphological observations and anatomy from the pre-pupal to adult stages. To discover the genes related to stinger development, we first comprehensively compared the stinger transcriptome at different stages and screened 1282, 186, and 166 highly expressed genes in the stingers of 1- and 5-day-old worker pupae and newly emerged worker bees (NEBs), respectively, then identified 25 DEGs involved in the early stage of stinger development. We found that *Dll* was a key candidate gene in the early development of *A. mellifera* stingers by combining analyses of the protein–protein interaction network and spatiotemporal expression patterns. An RNAi experiment showed that about 20% of individuals exhibited tip bending in the piercing parts of their stingers in the *Dll*-dsRNA-treated group, with the morphology presenting as side–side or front–back tip bending. This indicates that *Dll* plays a vital role in the early development of *A. mellifera* stingers. Together, our study provides insight into the molecular mechanism of Hymenoptera stinger development and an inspiration for the molecular breeding of gentle honeybee species with stinger abnormalities.

## 1. Introduction

Honeybees are not only important model organisms for the development of Hymenoptera insects, but are also well-known pollinators in nature. As they live in a complex environment, honeybees face a variety of threats from small arthropods, such as wasps, spiders, and ants, and also are attacked by large predators such as humans and bears [1]. In order to defend their nest and brood and to store food, they can quickly prime the organization of defensive responses, such as pursuit and stinging. The stinger, which is an evolutionary modification of the ovipositor, is an important defensive weapon, and its primary function is to deliver venom to an attacker [2,3].

The honeybee stinger is about 2.5 mm in length and comprises two lancets, a stylet and a principal motor apparatus (a quadrate plate, oblong plate, and triangular plate) [4]. Using a tongue and groove mechanism, the lancets can slide freely on the two rails of the stylet in a reciprocating manner to pierce and penetrate tissue and seep venom through the seam between the lancets [5,6]. The lancets and stylet all have hollow structures, and their main chemical component is chitosan, which contributes to their superior mechanical properties, including a high elastic modulus and toughness [5,7]. Although the lancets and stylet both possess rearward-facing barbs, those of the lancets are much more pronounced. Each lancet bears about 10 barbs with a length of 11.3 to 17.6 μm and a distance of 20.6 to 94.3 μm; the barbs on the stylet are quite variable in size and number [1,8]. These barbs can help reduce the penetration force and prevent prey from slipping off the stinger during insertion. Moreover, the lancets and stylet have campaniform sensilla on their surfaces that can perceive afferent signals and trigger the stinging response of *A. mellifera* [9].

Owing to its elegant structures and superior mechanical properties, the stinger is torn from the honeybee’s body and embedded in the victim’s skin, continuing to inject venom after the initial sting [10]. Moreover, the delivery rate of venom from a honeybee stinger is fast: at least 90% of venom is delivered within 20 s, and it is completely exhausted after 1 min of separation [11,12]. During penetration, the honeybee stinger undergoes a helical and clockwise rotation, which helps to improve the puncturing process [13]. Meanwhile, the honeybee stinger possesses an ultrasharp sting tip and microscopic backward-facing barbs for self-defense, providing an exquisite example of a biomicroneedle for the further design of microneedles. Compared to an acupuncture microneedle, the penetration force of a stinger (*Apis cerana*) is only 5.75 mN, which is approximately one order of magnitude smaller, while the pull-out force (113.5 mN) is approximately 70 times larger, suggesting that stingers are a promising type of painless microneedle with a minimal insertion force and high soft-tissue adhesion [8].

To date, there have been numerous studies focused on the anatomy, constituents, mechanical properties, and biology of the stingers of honeybees. However, the key genes and molecular mechanisms involved in stinger development remain elusive. In this study, we first investigated the transcriptome of the *Apis mellifera* stinger at different developmental stages; identified the genes involved in the early, middle, and late stages of stinger development; and employed RNAi to disrupt the key candidate gene *Dll*. Our results demonstrated that *Dll* regulates stinger development in *A. mellifera*, which provides insight into the molecular mechanism of Hymenoptera stinger development and inspiration for the molecular breeding of gentle honeybee species with stinger abnormalities.

## 2. Results

### 2.1. Morphological Observations of Stingers at Different Developmental Stages

**Figure 1:**
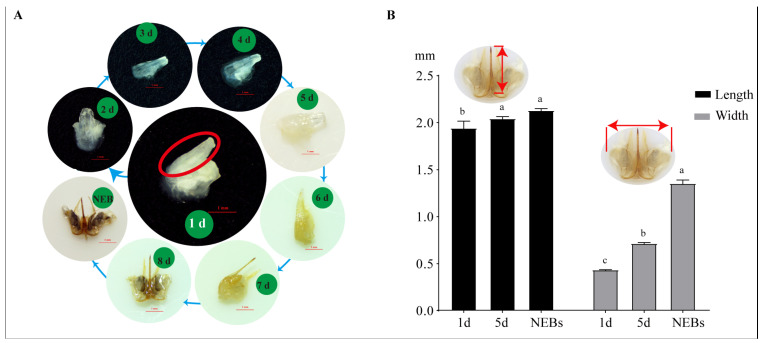
Morphology of worker stingers at the different development stages of *Apis mellifera*: (**A**) developmental changes in stingers from the pupal to adult stages, including 1- to 8-day-old pupae and newly emerged bees (NEBs); (**B**) length and width of stingers from 1- and 5-day-old pupae and NEBs. Red ellipses represent the piercing parts. Data are shown as the mean ± SEM (*n* = 45) for each group. Different lowercase letters above bars indicate significant differences among different groups.

Based on morphological observations and anatomy from the pre-pupal to adult stages (Figure S1), we could not detect stinger-like tissue during the pre-pupal stage, but found stinger tissue at the terminal abdominal segment of 1-day-old worker pupae, indicating that the earliest point for the appearance of stingers in bees is at the 1-day-old worker pupal stage. Furthermore, the morphology of stingers at different stages showed that the stingers of 1- to 4-day-old worker pupae had almost similar features (Figure 1A): they were milky white in color, swollen and smooth due to containing a high level of liquid content, and had a velvety texture. The distal end and sheath of stingers from 5-day-old pupae were slightly wrinkled and beginning to color, with the entire tissue manifesting a yellowish appearance. The stinger apparatuses of 6-day-old pupae had a striking yellow color and were further shrunk, with the piercing parts becoming more elongated. Stingers gradually matured at the 7-day-old pupal stage: at this time, the piercing parts became thinner and the hardness was increased, with a pair of yellowish accessory plates. After emergence, stingers manifested a brown color.

The average lengths of the stingers of 1- and 5-day-old pupae and NEBs were 1.945 mm, 2.046 mm, and 2.132 mm, respectively, which gradually increased with age (Figure 1B). The widths of the stingers also showed a similar trend with age: 1- and 5-day-old pupae and NEBs showed average widths of 0.436 mm, 0.717 mm, and 1.355 mm, respectively, indicating that the widths of NEBs’ stingers were significantly larger than those of the other stages (Figure 1B).

### 2.2. Statistics and Validation of Transcriptome Data

**Figure 2:**
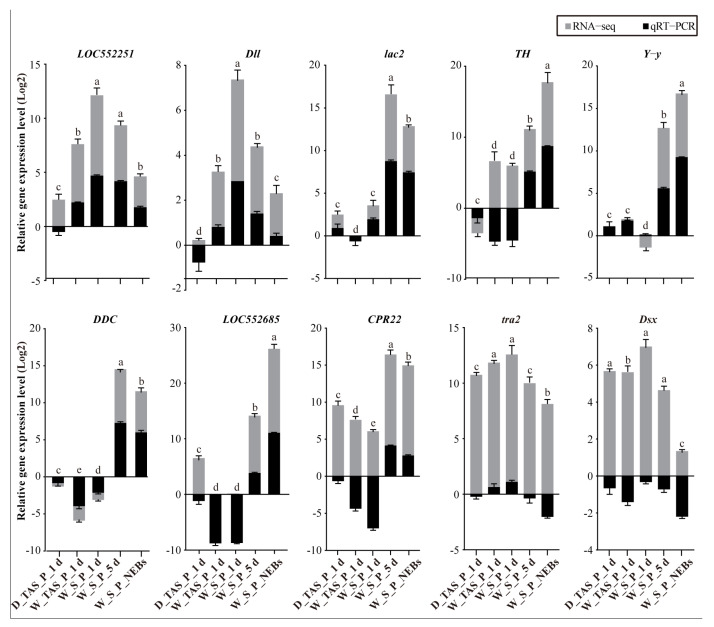
Validation transcriptome data using qRT-PCR. Data in the figure are the mean ± SD. Different lowercase letters above bars indicate significant differences among different groups.

After sequencing and filtering, 113.1 Gb of clean data were obtained from 15 specimens, with Q30 > 93% and mapping ratios > 87.7% (Table S1). Sequencing data showed that 11,140, 10,886, 10,823, 11,222, and 11,288 genes were expressed in the stingers of 1-day-old worker pupae (W_S_P_1 d), the stingers of 5-day-old worker pupae (W_S_P_5 d), the stingers of NEBs (W_S_NEBs), the terminal abdominal segments of 1-day-old worker pupae (W_TAS_P_1 d), and the terminal abdominal segments of 1-day-old drone pupae (D_TAS_P_1 d), respectively. To verify the reliability of the transcriptome data, ten genes, including *LOC552251*, *Dll*, *lac2*, *TH*, *Y-y*, *DDC*, *LOC552685*, *CPR22*, *tra2*, and *Dsx*, were randomly selected and validated with samples of W_S_P_1 d, W_S_P_5 d, W_S_NEBs, W_TAS_P_1 d, and D_TAS_P_1 d using qRT-PCR. The results showed that they had similar expression profiles with the transcriptome data (Figure 2), suggesting that the sequencing data were reliable for subsequent analysis.

### 2.3. Highly Expressed Genes in Stingers at Different Developmental Stages

**Figure 3:**
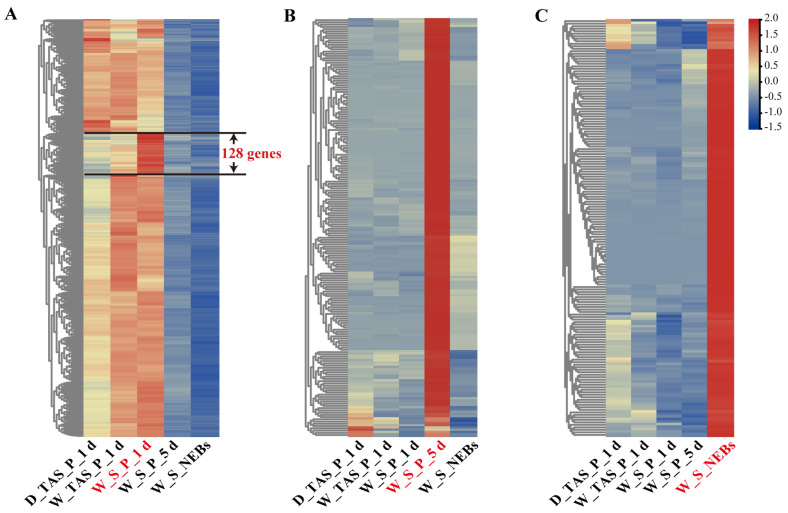
Clusters of the highly expressed genes in *Apis mellifera* stingers of 1-day-old worker pupae (**A**), 5-day-old worker pupae (**B**), and newly emerged worker bees (**C**).

Based on the screening criteria of highly expressed genes, we screened 1282 highly expressed genes in the stingers of 1-day-old worker pupae (W_S_P_1 d), with a lower expression in the stingers of 5-day-old worker pupae and newly emerged worker bees (Figure 3A and Table S2). Noteworthily, among them, there were 128 specific high-expression genes in W_S_P_1 d, while there was weak expression or no expression in the remaining four samples (W_S_P_5 d, W_S_NEBs, W_TAS_P_1 d, and D_TAS_P_1 d), including *distal-less* (*Dll, LOC726710*), *doublesex* (*LOC725126*), and *cuticular protein 16* (*LOC100576341*) (Table S3). According to the log_2_ (fold change) of W_S_P_1 d vs. W_S_P_5 d and W_S_P_1 d vs. W_S_NEBs from large to small, the top 20 common genes are listed in Table S4, including *proclotting enzyme* (*LOC552672*), *cuticular protein 16* (*LOC100576341*), *protein Wnt*-*1* (*LOC413502*), *tachykinin* (*LOC406083*), *LIM/homeobox protein Awh* (*LOC725574* and *LOC725532*), *BTB/POZ domain protein Ktl* (*LOC725415*), and *homeobox protein invected* (*LOC100577365*).

Under the same criteria, we screened 186 highly expressed genes in W_S_P_5 d, and a clustering analysis showed that their expression was more than twice as high as that in W_S_P_1 d and W_S_NEBs, including ten cuticle proteins (*LOC726950*, *LOC725089*, *LOC724382*, *LOC113218932*, *LOC409345*, *LOC726451*, *LOC724556*, *LOC107964828*, *LOC724649*, and *LOC102653988*), two melanin-synthesis-related genes (*laccase 2* and *yellow-h*), *chemosensory protein 6* (*LOC725103*), *period circadian protein* (*LOC406112*), and *putative defense protein 3* (*LOC726072*) (Figure 3B and Table S5). Meanwhile, we listed the top 20 most common genes based on the log_2_ (fold change) of W_S_P_5 d vs. W_S_P_1 d and W_S_P_5 d vs. W_S_NEBs from large to small (Table S6), which contained three genes related to resilin: *pro-resilin* (*LOC409176*) and *pro-resilin-like* (*LOC725019* and *LOC102654371*), and seven genes related to cuticle development: *pupal cuticle protein 20* (*LOC726950*), *cuticular protein 17* (*LOC724556*), *cuticular protein* (*LOC725089*), *pupal cuticle protein* (*LOC113218932*), *cuticle protein 64*-*like* (*LOC102653988*), *endocuticle structural glycolprotein ABD*-*4* (*LOC726995*), and *loricrin* (*LOC409962*).

Furthermore, a clustering analysis displayed that 166 genes were highly expressed in W_S_NEBs, with a weak expression or no expression in W_S_P_1 d and W_S_P_5 d, including five bee-venom-synthesis-related genes (*LOC406130*, *LOC551077*, *LOC550671*, *LOC406141*, and *LOC406145*), six cuticle-protein-related genes (*LOC551367*, *LOC727161*, *LOC409716*, *LOC552685*, *LOC100577189*, and *LOC413115*), and three melanin-synthesis-related genes (*tyrosine hydroxylase*, *dopamine N*-*acetyltransferase*, and *yellow*-*f*) (Figure 3C and Table S7). Based on the log_2_ (fold change) of W_S_NEBs vs. W_S_P_1 d and W_S_NEBs vs. W_S_P_5 d from large to small, the top 20 most common genes are listed in Table S8, including two Hymenoptera-specific cuticular proteins, *apidermin 1* (*LOC551367*) and *apidermin 3* (*LOC409716*), and three genes associated with bee venom, *melittin* (*LOC406130*), *phospholipase A2* (*LOC406141*), and *secapin* (*LOC406145*).

### 2.4. Identification of DEGs Associated with Stinger Development

**Figure 4:**
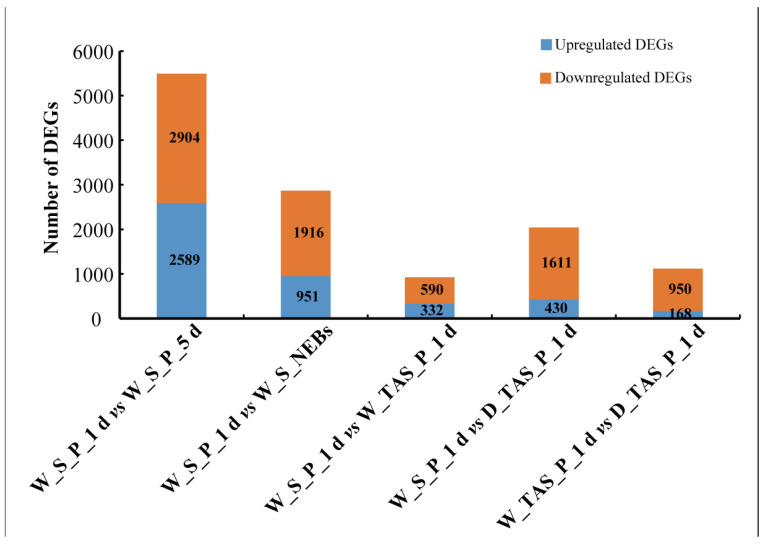
Statistics of differentially expressed genes (DEGs) among different stinger samples.

As 1-day-old worker pupae are in the earliest stage where the appearance of stingers occurs, we compared W_S_P_1 d with the other four groups and identified 5493, 2867, 922, and 2041 differentially expressed genes (DEGs) in the groups of W_S_P_1 d vs. W_S_P_5 d, W_S_P_1 d vs. W_S_NEBs, W_S_P_1 d vs. W_TAS_P_1 d, and W_S_P_1 d vs. D_TAS_P_1 d, respectively (Figure 4). There were 1118 DEGs between W_TAS_P_1 d and D_TAS_P_1 d.

**Figure 5:**
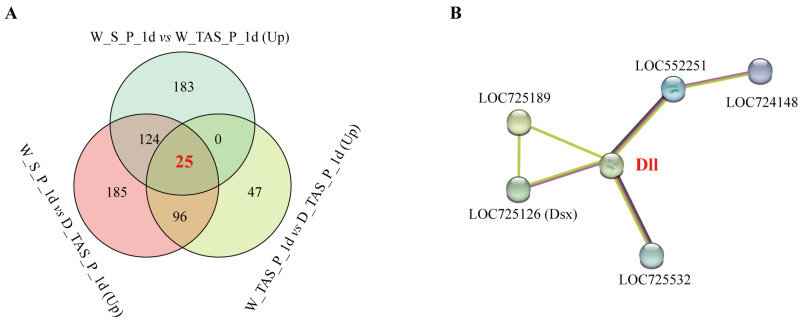
Identification of DEGs associated with stinger development: (**A**) Venn diagram of upregulated DEGs from W_S_P_1 d vs. W_TAS_P_1 d, W_S_P_1 d vs. D_TAS_P_1 d, and W_TAS_P_1 d vs. D_TAS_P_1 d; (**B**) protein–protein interaction of the Dll gene from the common upregulated DEGs of W_S_P_1 d vs. W_TAS_P_1 d and W_S_P_1 d vs. D_TAS_P_1 d.

To identify the DEGs involved in stinger development, 25 common upregulated DEGs from W_S_P_1 d vs. W_TAS_P_1 d, W_S_P_1 d vs. D_TAS_P_1 d, and W_TAS_P_1 d vs. D_TAS_P_1 d were chosen as candidate genes, including six transcription factors: *distal*-*less* (*Dll, LOC726710*), *LIM/homeobox protein Awh* (*LOC725532* and *LOC725574*), *homeobox protein aristaless* (*LOC552251*), *homeobox protein MSX-2* (*LOC724148*), and *transcription factor AP*-*2*-*epsilon* (*LOC410893*) (Figure 5A and Table S9). In order to investigate the protein–protein interaction network, 149 common upregulated DEGs from W_S_P_1 d vs. W_TAS_P_1 d and W_S_P_1 d vs. D_TAS_P_1 d were subjected to STRING, which predicated that Dll was a hub gene that could interact with five DEGs, including protein LIM/homeobox protein Awh (LOC725532), homeobox protein aristaless (LOC552251), homeobox protein MSX-2 (LOC724148), doublesex (Dsx, LOC725126), and bric-a-brac 2 (LOC725189). Taken together, we considered that Dll was a key gene for stinger development in *A. mellifera*.

**Figure 6:**
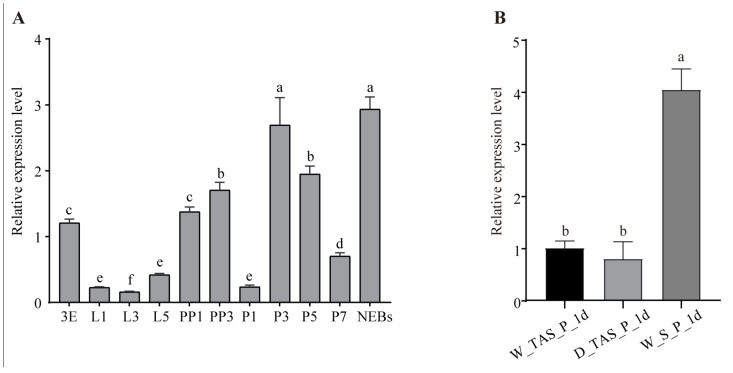
Temporal and spatial expression profiles of the *Apis mellifera Dll* gene. (**A**) Stage expression patterns of *Dll* at different development stages: 3E: 3-day-old egg; L1, L3, and L5: 1-, 3-, and 5-day-old larvae; PP1 and PP3: 1- and 3-day-old pre-pupae; P1, P3, P5, and P7: 1-, 3-, 5-, and 7-day-old pupae, respectively; NEBs: newly emerged bees. (**B**) Tissue expression patterns of *Dll* in *A. mellifera*: W_TAS_P_1 d: 1-day-old worker’s terminal abdominal segment (no stinger); D_TAS_P_1 d: 1-day-old drone’s terminal abdominal segment; W_S_P_1 d: stingers of 1-day-old worker pupae. Data in the figure are the mean ± SEM. Different lowercase letters above bars indicate significant differences among different groups.

A SMART prediction suggested that Dll harbors a well-conserved homeobox (HOX) domain. Stage expression patterns exhibited fluctuations in *Dll* at different developmental stages (Figure 6A): It had higher expression in 3-day-old eggs and decreased significantly in 1-day-old larvae, with the lowest expression in 3-day-old larvae. After that, it showed an increased and then decreased tendency, with a higher expression in 3-day-old pre-pupae and 3-day-old pupae and the highest peak in newly emerged worker bees. These profiles suggest that *Dll* might play a distinct role in stinger development at the early, middle, and late stages. To determine whether *Dll* was heavily expressed in W_S_P_1 d, its expression was investigated in W_S_P_1 d, W_TAS_P_1 d, and D_TAS_P_1 d using qRT-PCR, which displayed that *Dll* was significantly highly expressed in W_S_P_1 d compared to W_TAS_P_1d and D_TAS_P_1d (Figure 6B). These results further suggest that *Dll* genes might play an important role in the development of stingers.

### 2.5. Expression Profiles of Genes Involved in Sex Determination in Stingers at Different Stages

**Figure 7:**
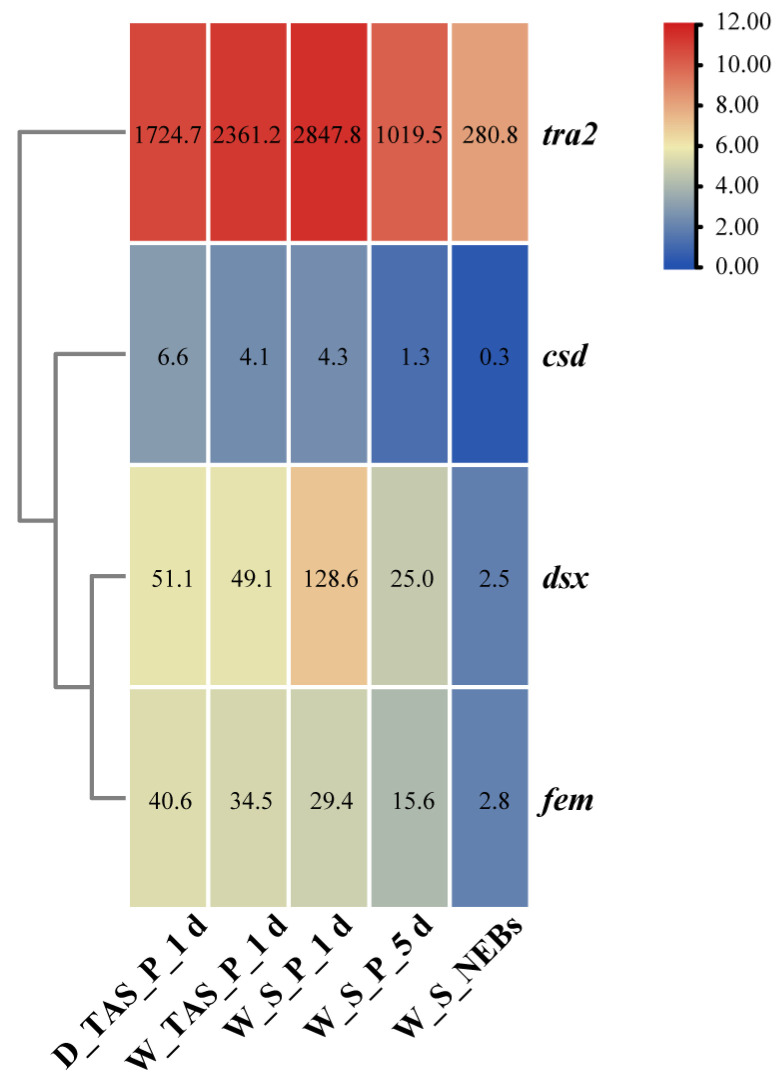
Expression profiles of four genes associated with sex determination in *Apis mellifera*.

As the stingers of *A. mellifera* evolved from ovipositors (female reproductive organs), the expression patterns of four genes (*tra2*, *dsx*, *csd*, *fem*) related to sex determination were investigated in the transcriptome data of W_S_P_1 d, W_S_P_5 d, W_S_NEBs, W_TAS_P_1 d, and D_TAS_P_1 d (Figure 7). They showed the highest expression in W_S_P_1 d and then gradually decreased with age, suggesting that these genes might play a critical role in stinger development. Among them, *tra2* had higher expression in W_S_P_1 d (FPKM = 2847.8), W_S_P_5 d (FPKM = 1019.5), W_S_NEBs (FPKM = 280.8), W_TAS_P_1 d (FPKM = 2361.2), and D_TAS_P_1 d (FPKM = 1724.7); *dsx* displayed more than twice the expression in W_S_P_1 d compared to W_TAS_P_1 d and D_TAS_P_1 d; and *csd* was weakly expressed in all samples.

### 2.6. Dll Knockdown Lead to Tip Bending of the Piercing Parts of Stingers

**Figure 8:**
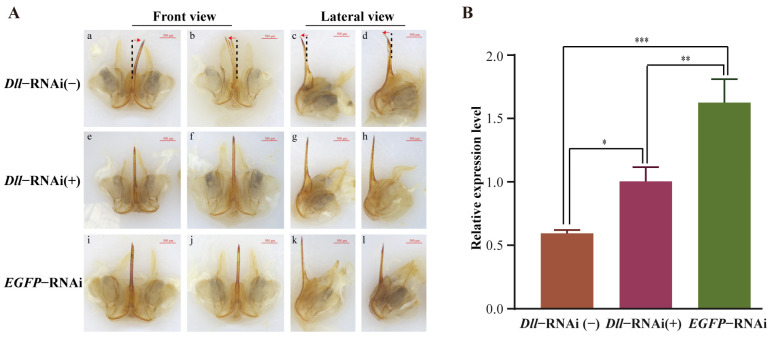
Stinger development in the *Dll* repression analysis of newly emerged worker bees (NEBs). (**A**) Photographs of a representative stinger from dsRNA (*Dll* or EGFP)-treated, newly emerged workers: (**a**,**b**) front view of a stinger from *Dll*-dsRNA-treated individuals with tip bending from side to side; (**c**,**d**) lateral view of a stinger from *Dll*-dsRNA-treated individuals with tip bending from back to front; (**e**–**h**) morphology of a stinger from *Dll*-dsRNA-treated individuals without tip bending; (**i**–**l**) morphology of a stinger from EGFP-dsRNA-treated individuals. A red arrow indicates the bending direction of the stinger from *Dll*-dsRNA-treated individuals. (**B**) Relative expression of the *Dll* gene in stingers from individuals with tip bending: *Dll*-RNAi(-): stingers from NEBs manifested the phenotype of tip bending when their larvae were treated with *Dll*-dsRNA; *Dll*-RNAi(+): stingers from NEBs manifested a normal phenotype when their larvae were treated with *Dll*-dsRNA; EGFP-RNAi: stingers from NEBs treated with *Dll*-dsRNA during larval stages. Data are presented as the mean ± SEM, and the significance of differences was assessed by an ANOVA using SPSS 8.0. Asterisks above bars indicate significant differences among different groups (* *p* < 0.05, ** *p* < 0.01, *** *p* < 0.001).

After emergence, approximately 20% of individuals exhibited tip bending of the piercing parts of stingers in the *Dll*-dsRNA-treated group, while all of the EGFP-dsRNA-treated group possessed a normal straight stinger phenotype (Table S10). In the *Dll*-dsRNA-treated group, stingers with tip bending displayed a different morphology, including tip bending from side to side (Figure 8(Aa,Ab)) and back to front (Figure 8(Ac,Ad)). Expression patterns showed that *Dll* was decreased significantly in the stingers of the *Dll*-dsRNA-treated group, especially those stingers with tip bending, which exhibited the lowest expression (Figure 8B). These results demonstrate that *Dll* plays an important role in the development of *A. mellifera* stingers.

## 3. Discussion

Hymenopteran insects, including the *Apidae* (bees), *Vespidae* (wasps, yellow jackets, hornets), and *Formicidae* (specifically, fire ants) species, have specific stingers that evolved from ovipositors and act as defense organs [14]. However, the molecular mechanisms underlying stinger development remain elusive. To our knowledge, this is the first study to report a comparative transcriptome analysis of the *A. mellifera* stinger at different stages. Furthermore, our results demonstrate that knockout of the expression of *Dll* gave rise to tip bending of the piercing parts of the stinger, suggesting that it plays a critical role in *A. mellifera* stinger development.

Combining morphological observation and anatomy from the pre-pupal to adult stages, we found that the 1-day-old worker pupal stage is the earliest point for the appearance of stingers in bees (Figure S1 and Figure 1). According to changes in the shape and color of the stinger, 1- and 5-day-old pupae and NEBs were chosen as the critical developmental stages. The terminal abdominal segments of 1-day-old worker/drone pupae (W_TAS_P_1 d and D_TAS_P_1 d) were used as the control group. Using comparative transcriptomic analysis, 25 DEGs related to stinger development were identified (Figure 5A). Of them, a STRING prediction showed that Dll is a hub gene that interacts with five genes (Figure 5B). It is reported that *Dll* has seven downstream target genes, including *bric a brac*, *spineless*, *aristaless*, *BarH1/BarH2*, *Dwnt5*, *disconnected*, and *Serrate* [15]; thus, we speculated that *homeobox protein aristaless* and *bric a brac 2* might be potential downstream targets for *Dll* in stinger development. In addition, three genes (*LIM/homeobox protein Awh*, *homeobox protein aristaless*, and *Dsx*) were highly expressed in the stingers of 1-day-old worker pupae (Table S2). *LIM/homeobox protein Awh* belongs to the LIM homeodomain (LIM-HD) family, which plays crucial roles in gonadal development. For example, the loss of the LIM-HD gene *Lhx2* gives rise to a disrupted tubular organization in mouse testes [16]; *Lhx9* is essential for mouse gonad formation [17], and an *Lhx8* deficiency affects mouse ovarian development, without a disruption of male gonadal development [18]. It is reported that *aristaless* also has essential roles in the gonadal development of mammalians. In mice, males manifested smaller testes and seminiferous tubules of a larger diameter when *aristaless* was knocked out [19]. In marsupials and mice, the expression of *aristaless* was detected in germ cells during spermatogenesis [20]. In *Drosophila*, *dsx* and its downstream *bric a brac*-regulated gonad stem cell niche development were found [21]. The above literature suggests that these genes play important roles in gonad development. Furthermore, because stingers are derived from female reproductive organs combined with *Dll* spatiotemporal expression patterns (Figure 6), we consider that this gene is a key candidate gene for stinger development.

In *Drosophila*, *Dll* has a prominent role in appendage development (such as antennae and legs) and the formation of the peripheral nervous system [15]. It also affects the development of melanic wing patterns across pierid and nymphalid butterflies [22]. To explore the physiological function of *Dll* in stinger development, the expression of *Dll* was knocked down using dsRNA mixed with artificial larval diets. After emergence, approximately 20% of individuals exhibited tip bending of the piercing parts of their stingers in the *Dll*-dsRNA-treated group (Table S10), with *Dll* expression being dramatically decreased compared to the *EGFP*-dsRNA-treated group (Figure 8B). This result indicates that *Dll* is an important gene for *A. mellifera* stinger development and provides a new insight for elucidating the molecular mechanism of Hymenoptera stinger development. However, there was a low penetrance of individuals with tip bending of their stingers in the *Dll*-dsRNA-treated group. We speculated that there are three possible reasons. Firstly, the dose of *Dll*-dsRNA mixed with artificial diets was low. The expression of *Dll* was significantly decreased in the stingers of *Dll*-dsRNA-delivered individuals, but most did not display an obvious phenotype (tip bending). This phenomenon may be because the level of gene silencing did not reach the threshold to induce stinger bending or because these individuals only experienced slight changes, such as an abnormality in the number or structure of barbs, which cannot be observed using an ordinary stereomicroscope. Secondly, *Dll* was knocked down at an early stage (2- to -4-day-old larvae), which resulted in a lower phenotypic rate in adults. Previous studies have shown that individuals administered dsRNA in their early life stages display a lower penetrance of knockdown phenotypes in their adult stage compared to those introduced to dsRNA by an intra-abdominal injection in adulthood [23,24]. Thirdly, the approach of orally delivering dsRNA affected RNAi efficiency. It was reported that dsRNA was degraded in the digestive systems of insects, which led to a lower sensitivity to RNAi when it was ingested using feeding [25].

Cuticle proteins play a critical role in the physical properties and function of cuticles [26]. In *Locusta migratoria*, the loss of *cuticle protein 8* affects the structure of the ovipositor [27]. We identified some cuticle proteins that were highly expressed in the stingers of 1- and 5-day-old pupae and NEBs (Tables S2, S5 and S7), suggesting that these genes might play a central role in the early, middle, and late stages of stinger development. During stinger development and tanning, its color changed from white to brown. The melanin-synthesis-related genes of the stingers of 5-day-old pupae and NEBs were heavily expressed, which was consistent with the variation in structure and color, indicating that these genes might be involved in pigmentation and sclerotization. In particular, bee-venom-synthesis-related genes, such as *melittin*, were highly expressed in the stingers of NEBs compared to 1- and 5-day-old pupae. Our data indicate that these above-mentioned genes have prominent roles in the complex process of *A. mellifera* stinger development.

This study has potential limitations. We found that *Dll* knockdown led to tip bending of the piercing parts of stingers, indicating that this gene plays a vital role in the early development of *A. mellifera* stingers. But whether workers with *Dll* knockdown can survive in a natural colony and whether these bees can sting normally is not clear. We will further our focus on these interesting topics. In addition, we also identified some key genes that are highly expressed at different stages of stinger development, such as cuticle protein and melanin/venom-synthesis-related genes. However, the physiological function of these genes during stinger development remains unknown. As there is an established genome-editing tool (CRISPR/Cas9) for *A. mellifera* [28,29], future work involving the functional knockout of the above candidate genes to create bees with abnormal stinger development, without tanning stingers, or without the expression of *melittin* will provide new insight into the molecular breeding of gentle bee species.

## 4. Materials and Methods

### 4.1. Collection of Bee Samples

Three *A. mellifera* colonies were raised in apiaries at the College of Bee Science and Biomedicine, Fujian Agriculture and Forestry University. To accurately collect worker samples of different developmental stages, queens were confined to an empty comb to control egg-laying time for 3 h according to our previous method [29], and then we obtained individuals of defined ages, including samples of 3-day-old eggs, 1-/3-/5-day-old larvae, 1-/2-/3-day-old pre-pupae, 1- to /8-day-old pupa, and newly emerged bees (NEBs). Meanwhile, a virgin queen was treated with CO_2_ to lay unfertilized eggs, and we then accurately captured 1-day-old drone pupae (D_P_1 d) following a previous method with minor modifications [30]. Briefly, we introduced a 10-day-old virgin queen into a newly established queenless colony, inserted one empty drone comb into the colony, and installed a queen excluder at the entrance of the hive to prevent the virgin queen from leaving. The virgin queen was gently placed in a 50 mL centrifuge tube and treated with CO_2_ for 5 min on two successive days. After the initial oviposition of the virgin queen, we controlled the egg-laying timing for 3 h via restricting the queen on the drone comb and collected specimens when they developed into 1-day-old pupae.

### 4.2. Morphological Observation of A. mellifera

To monitor the earliest appearance stage of stingers, we dissected stingers at the terminal abdominal segment of each individual from the pre-pupal stage to NEBs under a stereomicroscope and took photographs. Twenty individuals at each stage were dissected as a biological replication.

Based on the observation of stingers at different development stages, three critical stages with obvious morphological differences were used to measure the longitudinal length and width of stingers. Fifteen stingers were dissected at each developmental stage, with three biological replicates.

### 4.3. RNA Extraction and cDNA Synthesis

Trizol was used to extract total RNA from the above-mentioned samples, and the synthesis of cDNA was carried out with a 5× HiScript II qRT Super Mix II kit (Vazyme Biotech, Nanjing, China) according to the manufacturer’s instructions. Finally, the concentration and purity of cDNA was determined by agarose gel electrophoresis and Agilent 5400 (Agilent, Santa Clara, CA, USA).

### 4.4. cDNA Library Construction and Sequencing

For sequencing, 20 stingers or 10 terminal abdominal segments were obtained at different stages, and their RNA was extracted. A total of 15 samples (with three replicates) were subjected to library construction, including the stingers of 1-day-old worker pupae (W_S_P_1d), the stingers of 5-day-old worker pupae (W_S_P_5d), the stingers of newly emerged worker bees (W_S_NEB), the terminal abdominal segments of 1-day-old worker pupae (W_TAS_P_1d), and the terminal abdominal segments of 1-day-old drone pupae (D_TAS_P_1d). Transcriptome sequencing was performed using an Illumina NovaSeq 6000 platform with 150 bp paired-end reads. The raw sequence data were deposited in the Genome Sequence Archive [31] in the National Genomics Data Center (https://ngdc.cncb.ac.cn/gsa/) (accessed on 23 June 2024), China National Center for Bioinformation/Beijing Institute of Genomics, Chinese Academy of Sciences (GSA: CRA017846).

### 4.5. Transcriptome Data Analysis

After being filtered, the clean reads were mapped to the reference genome of *Apis mellifera* (Amel_HAv3.1) using HISAT. To calculate the gene expression level, the FPKM method was employed via StringTie software (v2.1.5) [32], which was used to build protein–protein interaction networks of specially upregulated genes in W_S_P_1 d.

### 4.6. Quantitative Reverse Transcriptase Polymerase Chain Reaction (qRT-PCR)

qRT-PCR was carried out using a CFX384 Touch Real-Time PCR Detection System (Bio-Rad, Hercules, CA, USA), following the instructions of ChamQ SYBR Color qPCR Master Mix (Vazyme Biotech, Nanjing, China), with three biological replicates and three technical replicates for each trial. Each reaction system (10 μL) contained 2× ChamQ SYBR Color qPCR Master Mix 5 μL, cDNA 1 μL, forward and reverse primers of 0.2 μL each, and ddH_2_O 3.6 μL. The qPCR reaction conditions were as follows: 95 °C 3 min, 95 °C 10 s, and 60 °C 30 s (40 cycles), with a determination of the melting curve: After initialization at 65 °C, the temperature rose by 0.5 °C to 95 °C every 5 s. *Apis mellifera* actin (NM_001185146.1) was used as the internal control gene, and the relative expression levels of each gene in the different samples were calculated by the 2^−ΔΔCt^ method [33]. The primers used in this study were designed using Primer Premier 5.0 and are listed in Table S11.

### 4.7. RNAi Experiment

The dsRNA of *Dll* (XM_016915684.2) and EGFP was synthesized according to the instructions of the T7 High Yield RNA Transcription Kit (Vazyme Biotech, Nanjing, China). Two-day-old worker larvae were grafted onto 48-well tissue culture plates, which were placed in an incubator with a temperature of 35 ± 0.5 °C and a humidity of 95 ± 1%, and fed an artificial diet containing 4 μg of dsRNA of Dll for four successive days. The composition and daily allowance of diets was based on Kaftanoglu et al.’s method [34]. In the control group, larvae were provided with the same diet as the experiment group, except that it contained an equivalent amount of dsRNA of *EGPF* instead of *Dll*. We checked all individuals daily and removed dead specimens. Larvae were reared until emergence to examine stinger development. After the pre-pupation, the temperature of the incubator was adjusted to 35 ± 0.5 °C and the humidity to 75 ± 1%. For clarification, stingers were dissected from NEBs and examined independently under a stereomicroscope.

### 4.8. Statistical Analysis

Data are presented as the mean ± SEM or mean ± SD, and significance analysis was conducted with a one-way analysis of variance (ANOVA) using SPSS 8.0. Different lowercase letters (or asterisks) above bars indicate significant differences among different groups (* *p* < 0.05, ** *p* < 0.01, and *** *p* < 0.001).

## 5. Conclusions

In this study, according to morphological observation and anatomy, we found that the earliest time point for the appearance of stingers in *A. mellifera* was at the 1-day-old worker pupal stage. A comprehensive transcriptome analysis showed that the numbers of highly expressed genes were 1282, 186, and 166 in the stingers of 1- and 5-day-old worker pupae and newly emerged worker bees (NEBs), respectively. Among them, cuticle protein and melanin/venom-synthesis-related genes were highly expressed at distinct stages of stinger development, providing new insight into the molecular breeding of gentle bee species. Furthermore, we identified 25 DEGs involved in the early development of stingers. More importantly, *Dll* knockdown led to tip bending in the piercing parts of stingers, suggesting that it plays a vital role in the early development of *A. mellifera* stingers. These results provide insight into the molecular mechanism of Hymenoptera stinger development and inspiration for the molecular breeding of gentle honeybee species with stinger abnormalities.

## Data Availability

The raw transcriptome sequence data have been deposited in the Genome Sequence Archive in the National Genomics Data Center, China National Center for Bioinformation/Beijing Institute of Genomics, Chinese Academy of Sciences (GSA: CRA017846) and are publicly accessible at https://ngdc.cncb.ac.cn/gsa (accessed on 23 June 2024).

## Supplementary Materials

The following supporting information can be downloaded at: https://www.mdpi.com/article/10.3390/ijms251910746/s1.
