# Supplementary figures and images for: Transcriptomic Analysis of Genes Associated with Stinger Development at Different Life Stages of Apis mellifera
Source: Int J Mol Sci. 2024 Oct 6;25(19):10746. doi: 10.3390/ijms251910746 (PMC11477386; doi:10.3390/ijms251910746)

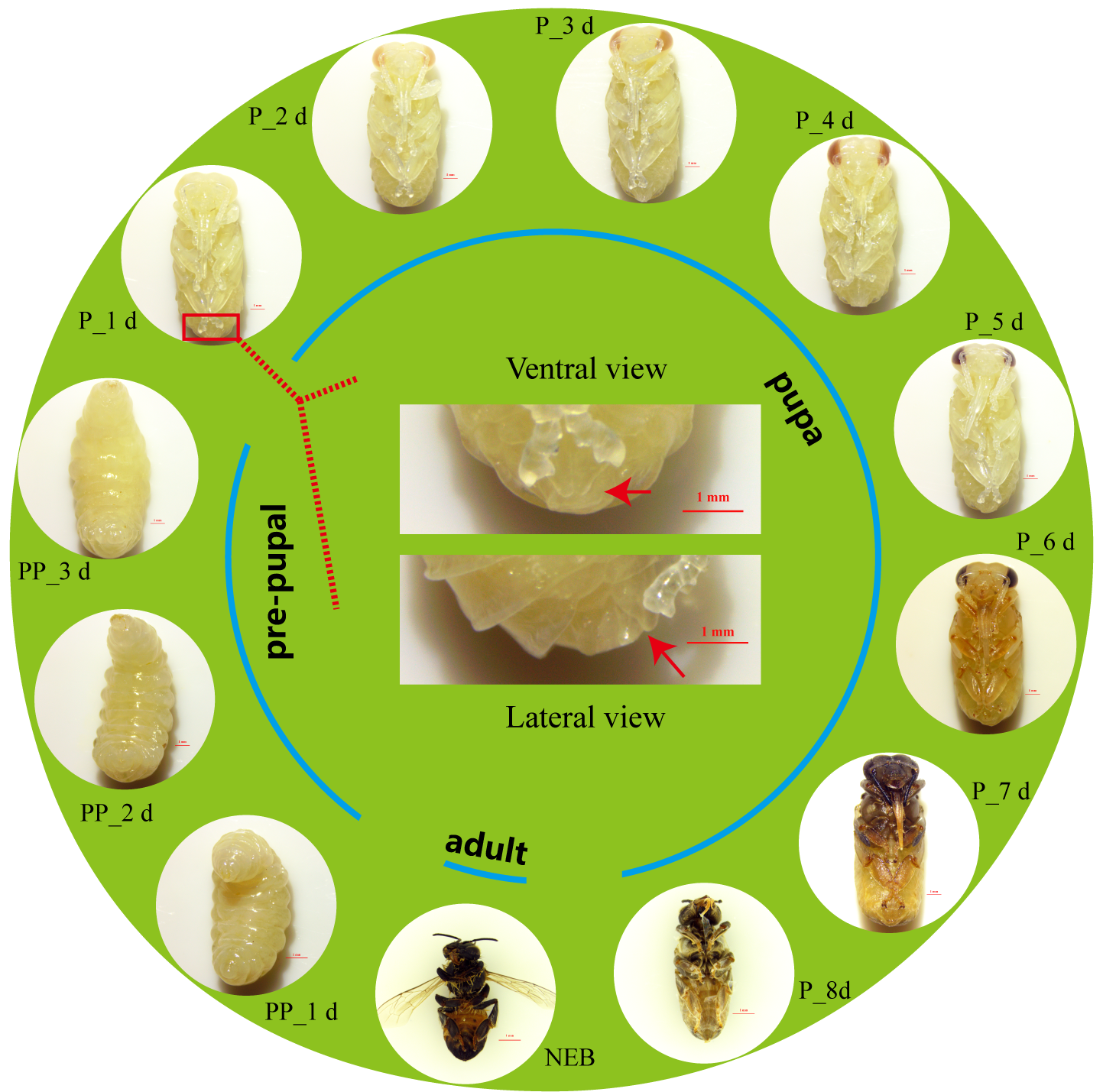

Supplement: Supplementary file 1 [file ijms-25-10746-s001.zip › Figure S1.tif]
